# Experimental Evaluation of Low Velocity Impact Properties and Damage Progression on Bamboo/Glass Hybrid Composites Subjected to Different Impact Energy Levels

**DOI:** 10.3390/polym12061288

**Published:** 2020-06-04

**Authors:** Ain Umaira Md Shah, Mohamed Thariq Hameed Sultan, Syafiqah Nur Azrie Safri

**Affiliations:** 1 Laboratory of Biocomposite Technology, Institute of Tropical Forestry and Forest Products (INTROP), UPM Serdang 43400, Selangor Darul Ehsan, Malaysia; snasafri@gmail.com; 2 Department of Aerospace Engineering, Faculty of Engineering, Universiti Putra Malaysia, UPM Serdang 43400, Selangor Darul Ehsan, Malaysia; 3 Aerospace Malaysia Innovation Centre (944751-A), Prime Minister’s Department, MIGHT Partnership Hub, Jalan Impact, Cyberjaya 63000, Selangor Darul Ehsan, Malaysia

**Keywords:** low velocity impact, hybrid composites, damage progression, glass fibre, bamboo

## Abstract

Six impact energy values, ranging from 2.5 J to 10 J, were applied to study the impact properties of neat epoxy and bamboo composites, while six impact energy values, ranging from 10 J to 35 J, were applied on bamboo/glass hybrid composites. Woven glass fibre was embedded at the outermost top and bottom layer of bamboo powder-filled epoxy composites, producing sandwich structured hybrid composites through lay-up and molding techniques. A drop weight impact test was performed to study the impact properties. A peak force analysis showed that neat epoxy has the stiffest projectile for targeting interaction, while inconsistent peak force data was collected for the non-hybrid composites. The non-hybrid composites could withstand up to 10 J, while the hybrid composites showed a total failure at 35 J. It can be concluded that increasing the filler loading lessened the severity of damages in non-hybrid composites, while introducing the woven glass fibre could slow down the penetration of the impactor, thus lowering the chances of a total failure of the composites.

## 1. Introduction

Generally, mechanical properties are the most important information that needs to be measured in materials [1]. However, in real life situations, impact is one of the very common phenomena experienced by all materials and structures. An instantaneous load applied on a surface during an impact event can cause unpredictable damages, which can sometimes lead to total structural failure. It is even worse if the low velocity impact event caused non-visible damages; the accumulated damages after several repeated events can lead to serious failures [2,3]. Experimentally, low velocity impact can be simulated using the drop test rig instrument. The Izod and Charpy impact testers are more descriptive of fracture toughness, which can be considered as the mechanical properties of materials [4].

It was cited from one source that low velocity impact is an impact event below 10 m/s, while intermediate, high and hypervelocity impacts correspond to the range of 10 m/s–50 m/s, 50 m/s–1000 m/s and 2 km/s–5 km/s respectively. Different ranges of impact velocities are very important to analyse, rather than saying that only high velocity needs more attention, as each structure has its own surroundings and working environment [5]. Different mechanisms of damage initiations can be observed being subjected to impact loading, and these observations illustrate the dissipation energy from the impact force to the materials [6].

Compared to metal materials, worse damages were experienced by fibre-reinforced composites as damages that could occur in a wider form, and which are matrix cracking, lamina splitting, fibre-matrix interfacial disbanding, and the last failing mechanism of fibre breakage. These situations lead to a complex analysis of composites compared to metals [7,8]. A study was specifically conducted to examine the effects of thickness, stacking sequence and scaling technique on the barely visible impact damage of composite laminates. Interlaminar damages in terms of delamination are one of the observations analysed using the ultrasonic phased array inspection technique in this study [9]. Further analyses on the impacted samples were conducted to determine the residual strength through compression after impact testing. It was in a good agreement, as samples impacted with a higher impact energy possessed a lower residual compressive strength due to the higher degree of damages that occurred [10]. An overall study on the impact properties of sugar palm/glass reinforced epoxy composites covered the impact testing, damage analysis using c-scan technique and post-impact properties through compression after impact testing. The damage area of sugar palm/glass hybrid composites increased proportionally with an increase in the impact energy levels [11]. Different parameters for stitched and unstitched flax fibre-reinforced epoxy composites were studied for their impact damages. The overall findings showed that delamination was not the main damage mode in both types of composites. Besides that, the propagation of in-plane cracks within the composites was not enhanced by stitching fibres compared to unstitched fibres [12].

The use of bamboo in structural applications as well as daily life utensils had been explored since a hundred years back. The light-weight advantage of natural bamboo culms, with a comparable strength to mild steel for some species, suits the current demand for producing environmentally friendly materials with acceptable properties comparable to conventional ones. The distinctive fast-growing features of bamboo plant make it among the most promising sources of fibre supplies in the continuous production of composites [13]. Similarly to any other natural fibres, different methods of extraction give different types of bamboo fibres with strength variations and, by some measures, lower strength properties compared to the bamboo culms [14].

Bamboo-Polyvinyl Chloride (PVC) composites were developed in an attempt to replace the wood-PVC composites. The inclusion of bamboo particles in PVC had significantly improved the flexural modulus of elasticity compared to neat PVC, which indirectly enhanced the potential of bamboo-PVC composites as a replacement to wood-PVC composites [15]. In a different study, increasing the concentration of tetraethyl orthosilicate (TEOS) in ethanol solution used to modify the bamboo flour slightly increased the shear modulus of bamboo/polyamide composites. However, the effects of different concentration of TEOS was not observed on the percentage of crystallinity of bamboo/polyamide composites through the differential scanning calorimetry analysis conducted in the similar study [16]. Adding 20% bamboo fibre in polypropylene (PP) increased all the tensile, flexural and impact strengths of bamboo/PP composites compared to neat PP. Further research with the inclusion of hollow glass microspheres in the bamboo/PP composites suggested that the hybrid composites can be used in light-weight and high-strength components in engineering applications [17].

Many studies have been conducted by researchers around the world to widen the use of bamboo fibres in composites, yet most of the studies have focused on the mechanical properties with only a small area covering the impact properties. The current study is the continuation of a previous study on the mechanical properties of bamboo/glass hybrid composites [18]. In the present study, the damage progressions on short bamboo fibre composites and bamboo/glass hybrid composites were analysed. The initiative of using bamboo powder as fillers in some measure filled the gap of utilising all forms of bamboo fibres in the development of bamboo composites.

## 2. Experimental Section

### 2.1. Materials

Epoxy matrix typed Epoxamite 100 with 103 slow hardeners were used in this study. Bamboo from the species of Bambusa vulgaris was collected from Raub, Pahang in Peninsular Malaysia, and the extraction process was carried out in several laboratories within Universiti Putra Malaysia. The preparation of bamboo powder was mentioned in the previous study [19]. Woven glass fibre-typed E 600 was chosen to be hybrid with bamboo powder, as it possesses a higher impact strength when compared to other types of glass fibre [20]. Table 1 lists the general properties of the epoxy matrix and E-glass fibres obtained from the suppliers.

### 2.2. Composite Fabrication

The neat epoxy, short bamboo fibre composites and bamboo/glass hybrid composites were fabricated as reported in the previous study [18]. The fabricated composites were listed as neat epoxy (EP), non-hybrid bamboo composites with 10% loading (EP-BF10) and 30% loading (EP-BF30), and bamboo /glass hybrid composites with 10% loading (EP/G-BF10) and 30% loading (EP/G-BF30).

### 2.3. Characterisation of Impact Properties

The low velocity impact was simulated using an instrumented drop weight impact test machine, model IMATEK IM10, at the Faculty of Engineering, Universiti Putra Malaysia. The impact testing was conducted with five repeatability samples. The instrument was equipped with IMATEK Impact Analysis software, to record and process the impact results data. A hemispherical tip striker with a radius of 5 mm attached to a variable weight resulting in a total weight of 5.101 kg was dropped from several desired heights onto the clamped sample. Different heights were used to represent different magnitudes of the impact energy applied onto the samples, based on the following equation:

(1) $$E_{I}=mgh$$

where *m* is the total mass of the impactor, 5.101 kg; *g* is the gravitational acceleration, 9.81 m/s^2^; and *h* is the height of the impactor.

The raw data of the force, time, displacement, velocity and energy absorbed by the samples were recorded and calculated by the installed software. Table 2 shows the impact energy applied on the EP, EP-BF composites and EP/G-BF composites.

### 2.4. Characterisation of Impact Damages

The dye penetrant method was applied to observe the damages on the impacted samples. The length of the matrix cracking was measured to characterise the damage propagation as the response to different impact energies on the EP-BF composites, while the area of damage was measured for the EP/G-BF composites [21].

## 3. Results and Discussion

### 3.1. Force Displacement Analysis

**Figure 1:**
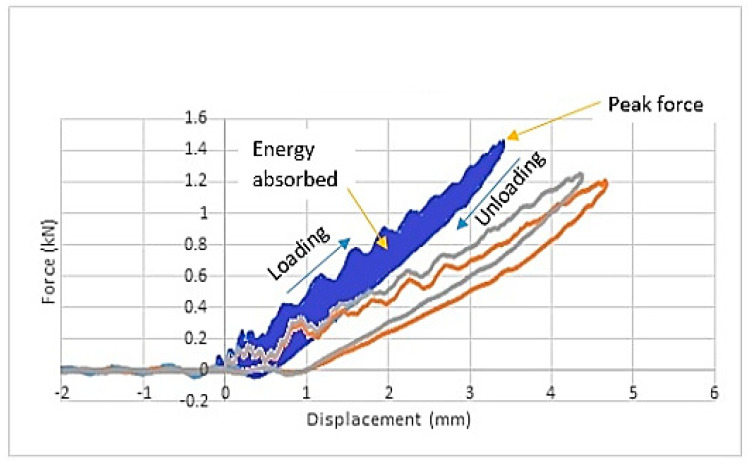
Force-displacement graphs of EP at the 2.5 J impact energy level showing closed curves.

Important information regarding the damage progression within the sample during an impact event can be obtained from the force-displacement graph. The movement of the impactor and the deformation of the impacted surface of the sample during contact with the impactor are marked as displacement values in the graph [22]. Figure 1 shows the force-displacement graphs of EP at the lowest impact energy level of 2.5 J.

The overlapping of the three graphs in the figure shows the repeatability of the three samples for EP under the same magnitude of impact energy. Testing conducted on all samples, for each impact energy level, was also repeated three times to confirm the repeatability of the results. The closed curve from the force-displacement graph indicates the non-full penetration damage of the sample tested, which indirectly explains that the full penetration of the impactor into the sample will produce an open curve in the force-displacement graph [23].

The ascending and descending parts of the closed curve explain the loading and unloading conditions, respectively. The ascending part also provides information about the impact bending stiffness of the samples. The greater the peak force, the stiffer the projectile-to-target interaction, thus shortening the contact period of the impactor onto the surface [24]. The relationship between a greater peak force and a stiffer target is a representation of a situation in which greater force is needed to initiate damage in stiffer materials. Besides the target stiffness, the peak force also depends on the magnitude of the impact energy from the impactor.

The peak deflection or peak deformation occurring in the sample is the value of the peak displacement from the force-displacement graph. In most graphs, the point of the peak deformation value is almost the same as the point for the peak force. However, it is clearly understood that the peak deformation can be identified as the turning point at which the force curve returns to zero after the loading condition or ascending curve, while the peak force is the maximum value in the vertical direction of the graph. It is clear that both the peak force and the peak deformation values were obtained from two different points [25].

**Figure 2:**
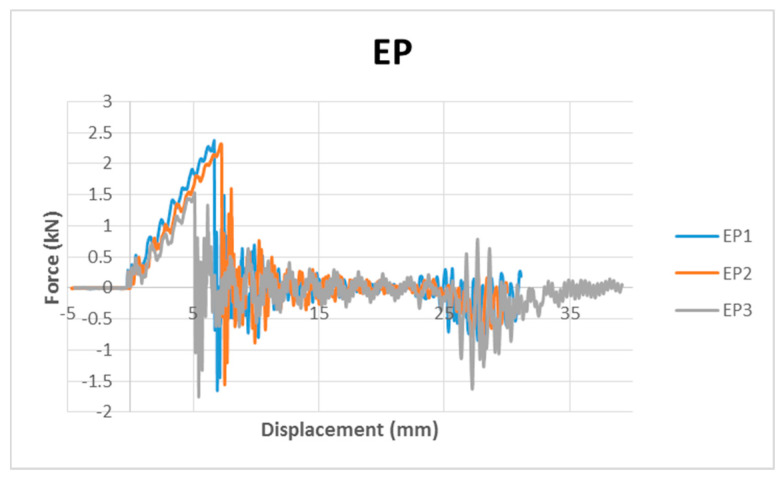
Force displacement graphs of EP at a 15 J impact energy level showing open curves indicating full penetration.

Another important value extracted from the force-displacement graph is the energy absorbed by the samples, which can be determined from the area under this graph. The energy absorbed is the kinetic energy transferred from the impactor to the samples during impact [24]. Both the energy absorbed throughout the impact event and the energy absorbed up to the peak deformation can be obtained from the force-displacement graph as shown in Figure 1. An example of a force-displacement graph for fully penetrated or perforated samples during impact can be seen in Figure 2.

The open curves show that the displacement increased monotonically with a decreasing force. The penetration of the samples demonstrates a situation where the force applied exceeded the maximum allowable force for the samples [24]. Throughout the low velocity impact analysis, the full penetration events will not be discussed. Therefore, the force displacement graphs of all the samples were first analysed to exclude the data for the penetrated samples. This explained the different maximum impact energy levels for each type of sample discussed in this study. The perfect overlapping graphs in Figure 2 confirmed the repeatability of the tests and the consistency of the samples. The excluded data for the broken samples was confirmed after these three repeatability tests.

It can be seen that different samples can withstand different maximum impact energy levels. Obviously, the second range of impact energy, as listed in Table 2, was higher when compared to the first range, as the inclusion of woven glass fibre was assumed to slow down the penetration of the impactor, thus increasing the maximum allowable force impacted on the surface of the composites. For all composites, the data analysed from the force-displacement graphs is presented in Table 3 and Table 4, which list the first and second ranges of impact energy applied on the samples, respectively. The relationship between the tabulated values will be further discussed in the following sections.

### 3.2. Peak Force Variation with Impact Energy

**Figure 3:**
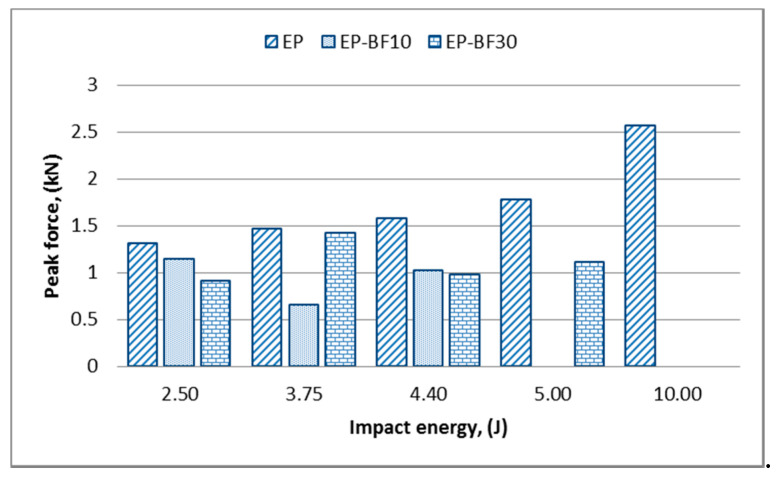
Variation of the peak force for the EP and EP-BF composites at the first range of impact energy levels.

Figure 3 shows the variation of the peak force for the EP and EP-BF composites at the first range of impact energy levels.

In Figure 3, it can be seen that the EP has the highest peak force when compared to EP-BF10 and EP-BF30 for all impact energy levels. This leads to the first conclusion that epoxy has the stiffest projectile-to-target interaction [24]. Comparing EP-BF10 and EP-BF30 gives a different trend at different impact energy levels. At an impact energy of 2.5 J and 4.4 J, the EP-BF10 shows a higher peak force compared to EP-BF30. However, at an impact energy of 3.75 J, the EP-BF10 has a lower peak force compared to EP-BF30 with a significant value. These inconsistent values were caused by the random orientation of the bamboo powder in the epoxy and the agglomeration of powder that might be occurring, which results from the poor distribution of the bamboo powder during fabrication [18].

**Figure 4:**
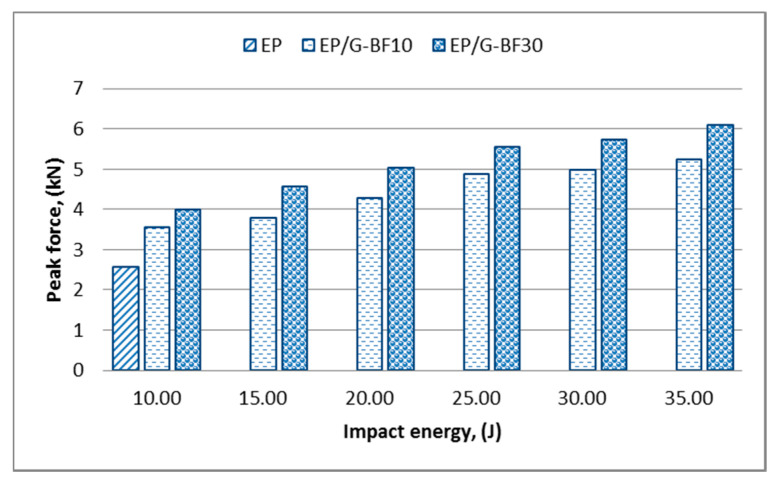
Variation of the peak force for the EP and EP/G-BF composites in the second range of impact energy levels.

Based on the first conclusion for epoxy, a comparison of the different loadings of bamboo composites made at impact energies of 2.5 J and 4.4 J is more consistent when compared to the comparison made at an impact energy of 3.75 J; these comparisons show that lower bamboo filler loading gives a stiffer projectile-to-target interaction [24]. However, at an impact energy of 5 J, the EP-BF30 could still withstand the force without breaking, while the EP-BF10 experienced total damage. This situation shows that EP-BF30 has good strength but lower stiffness, while the EP-BF10 shows the opposite trend. In the second range of impact energy levels, the hybrid composites respond consistently to the force applied, as illustrated in Figure 4.

The peak force increased as the impact energy increased for both loadings of hybrid composites. The inclusion of woven-type glass fibre on the outermost surface of the composites helps with a better force distribution when compared to the random orientation of fibre, resulting in a more consistent data trend [22]. The highest impact energy marked from the first range of impact energy levels in Figure 3, which was 10 J, was the lowest value in the second range of impact energy levels in Figure 4. At this impact energy, only the EP was comparable to the EP/G-BFC, while the EP-BFC samples failed and experienced total damage. From the values shown in both Figure 3 and Figure 4, the hybrid composites tend to have a higher peak force to initiate damages as expected. Moreover, woven-type glass fibres are good in impact resistant, and it is expected that a woven-type fibre of any material will have better resistance towards impact when compared to other types of fibre such as random and unidirectional fibres [26].

### 3.3. Energy Absorbed Variation with Impact Energy

The impact energy supplied during an impact event is converted into two fractions, which are the loss elastic energy and the energy absorbed by the sample. The absorbed energy is presented by the damage mechanisms on the sample or structure, where more severe damage can be an indication of more energy being absorbed [24,26]. The severity of the damage is subject to the mechanical properties of the reinforcement and the matrix, the shape of the impactor tip, the fibre orientation, the sample’s geometry and the impact energy levels [21].

Each sample absorbed a different amount of energy at each impact energy level, and thus a direct relationship of impact energy with the amount of absorbed energy was not advisable for explaining the severity of the damage on the samples. It is understood that a higher impact energy will cause a higher amount of impact energy to be absorbed, as depicted in Table 1 and Table 2 [25]. Therefore, the percentage of energy absorbed by each sample at the respective impact energy level presents a better relationship of the impact energy with the absorbed energy and explains the severity of damage on the samples.

**Figure 5:**
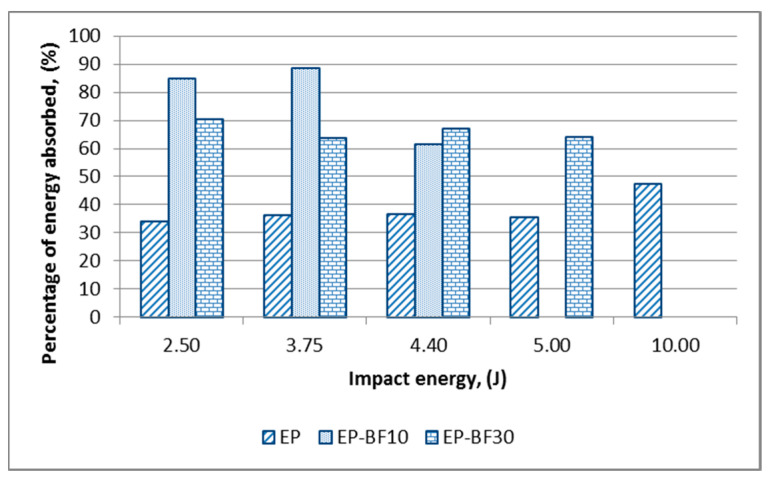
Percentage of energy absorbed by the EP and EP-BF composites at the first range of impact energy levels.

**Figure 6:**
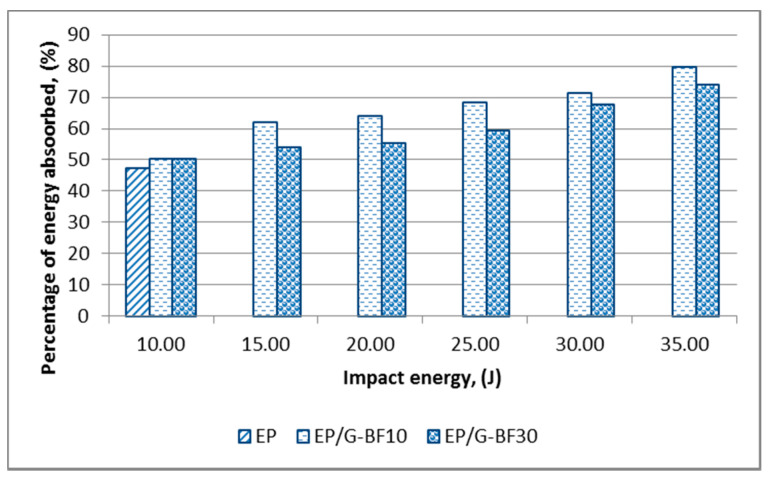
Percentage of energy absorbed by the EP and EP/G-BF composites at the second range of impact energy levels.

Figure 5 and Figure 6 present the percentage of energy absorbed by the EP, EP-BF and EP/G-BF composites, respectively.

**Figure 7:**
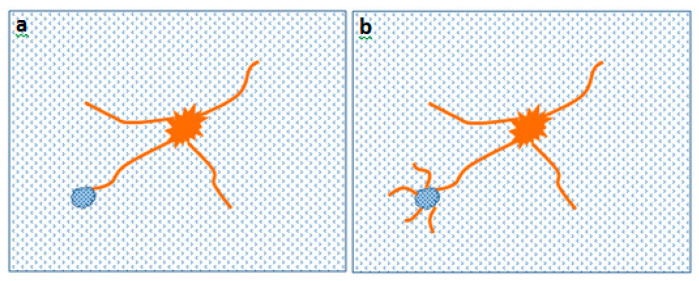
Agglomeration in bamboo composites can either (**a**) stop the damage progression or (**b**) cause more severe damage in the sample.

It was seen that an inconsistent trend was represented by the EP-BF composites compared to the EP in Figure 5. This is due to the random orientation of the short bamboo fibres, since the force being applied cannot be distributed evenly. At the same time, the agglomeration of bamboo powder that might be located within the structure tends to either slow down the damage propagation or worsen the damage by integrating a larger surface damage [8,13]. The inconsistent response of randomly oriented bamboo composites towards impact causes an inconsistent energy to be absorbed by the samples. This concept is illustrated in Figure 7.

Compared to non-hybrid composites, a clearer relationship between the percentage of energy absorbed and the different impact energy values of hybrid composites can be seen in Figure 6. As the impact energy increased, the percentage of energy absorbed increased. Besides this, at all impact energy levels, the percentage of energy absorbed for the EP/G-BF30 is lower than for the EP/G-BF10. This is in good agreement with the damage found on the samples, where EP/G-BF10 experienced more severe damage when compared to EP/G-BF30.

### 3.4. Damage Analysis on The Impacted Samples

**Figure 8:**
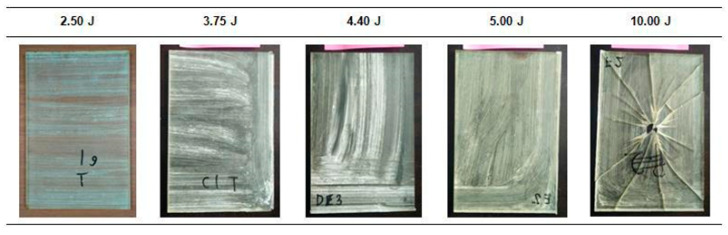
The EP samples after low velocity impacts at different impact energy levels.

The damage analysis will be separated based on the types of samples and their response towards different impact energy levels. Figure 8 shows the damages on the EP samples.

From the lowest energy values of 2.50 J to 5.00 J, the samples were free from cracks, and no dented surface was visible. However, at an impact energy level of 10.00 J, the EP sample was totally broken. This observation leads to the conclusion that neat polymer plates will experience total failure at a certain impact energy level without experiencing minor cracks [21]. This situation therefore lowers the dependency and safety of a product in real life applications. It might withstand a higher load, but an unexpected total failure might happen at any limit without any preliminary sign of damage.

**Figure 9:**
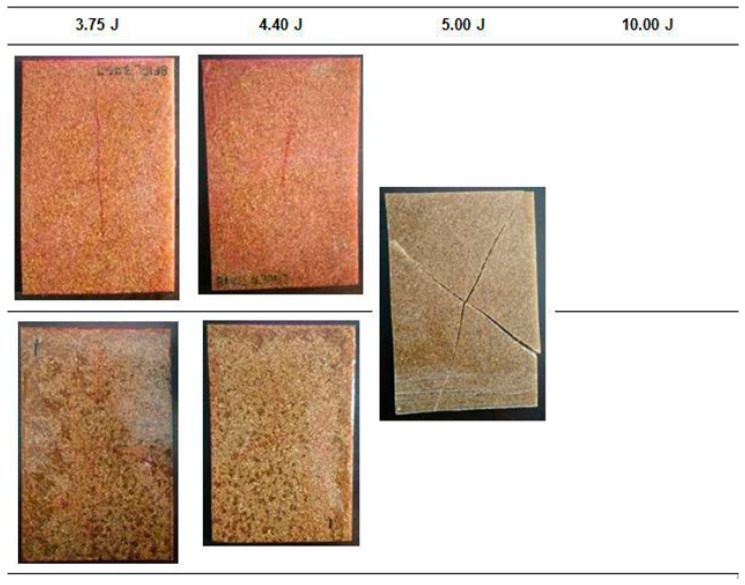
The damage on the impacted samples of EP-BF10 was observed with the help of the dye penetrant.

**Figure 10:**
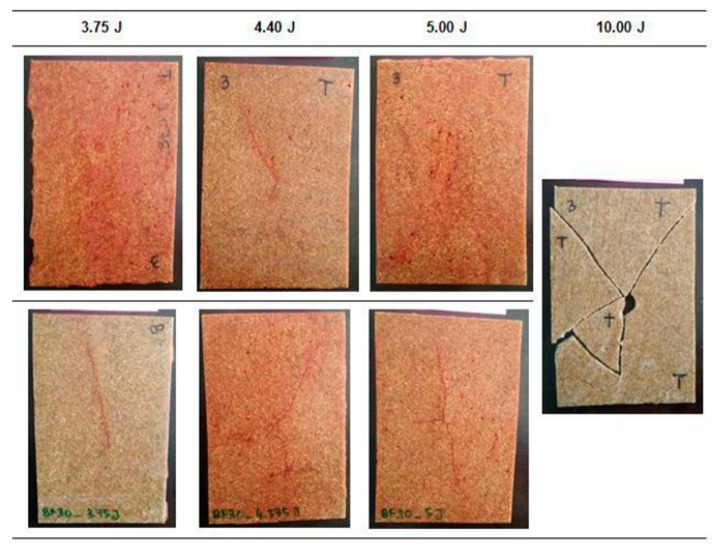
The damage on the impacted samples of EP-BF30 was observed with the help of dye penetrant.

The damage on the non-hybrid EP-BF composites after the low velocity impact was observed with the aid of the dye penetrant and is shown in Figure 9 and Figure 10.

As depicted in Figure 9, matrix cracking was detected on the EP-BF10 samples at 2.50 J, 3.75 J and 4.40 J impact energy levels. The matrix cracking propagates from the top surface to the bottom, and from the centre (where the impactor was dropped) to the sides of the rectangular plates. The EP-BF10 samples can withstand the first three impact energy levels; the propagation of matrix cracking stopped before reaching the sides of the samples. However, 10.00 J of impact energy enabled the matrix cracking to propagate until the end sides of the samples, thus breaking the rectangular plates into pieces. Compared to the visual look of the broken EP samples in Figure 9, the broken sample of EP-BF10 indicates a less severe damage as it broke into four large pieces that can be laid out, instead of numerous smaller pieces. Micro-sized fibres with a random orientation limit the analysis of the damage, as no trend can be suggested concerning the relationship of the impact energy levels with the severity of the damage caused [27].

Although no trend can be suggested regarding the propagation of matrix cracking, the analysis was presented in terms of the distance of the matrix cracking propagation from the centre to the side of the rectangular samples. It was found that the distance increased as the impact energy increased from 2.50 J to 4.40 J. These distances were measured on the bottom surface of the EP-BF10 samples, and the longest distance from the centre is recorded in Table 5.

Similar damage behaviour was observed on the EP-BF30 samples compared to the previous EP-BF10. Dye penetrant was used, and the observation of the damage is shown in Figure 10.

Matrix cracking was detected as propagating from the top to the bottom and from the centre to the sides of the impacted samples. However, small differences can be seen between the damage on the EP-BF30 and that on the EP-BF10, i.e., the number of lines of matrix cracking is lower than the number found on the EP-BF10. For the EP-BF30, only two obvious lines of matrix cracking were observed from the centre of impact, while in the EP-BF10 the damage propagated into four observable lines from the centre of impact. Figure 10 shows that the EP-BF30 withstood the 5.00 J impact energy but failed at 10.00 J, which is one level higher than for the EP-BF10. The damage analysis for the EP-BF30 is presented in Table 6.

The shorter distance of the damage propagation for the EP-BF30 suggested that increasing the bamboo fibre loading in the epoxy matrix improved the impact resistance of the composites. The impact resistance of the hybrid EP/G-BFC was expected to be higher compared to the non-hybrid EP-BF composites. The inclusion of woven glass fibres at the top and bottom outermost layers of the composites was believed to help in slowing down the impact absorption into the plates, thus reducing the damage [13].

**Figure 11:**
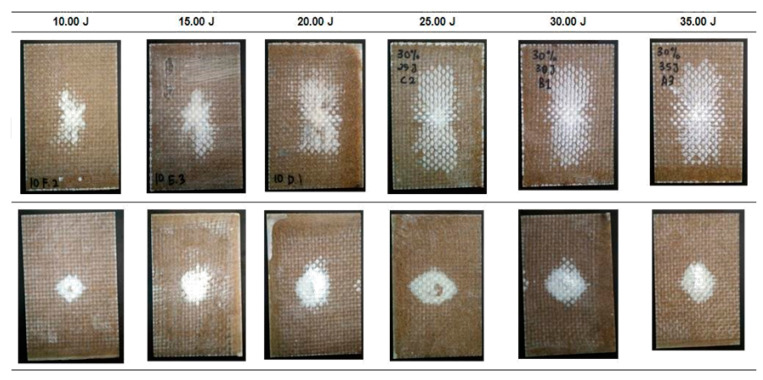
Visible damage on the impacted samples of EP/G-BF10.

**Figure 12:**
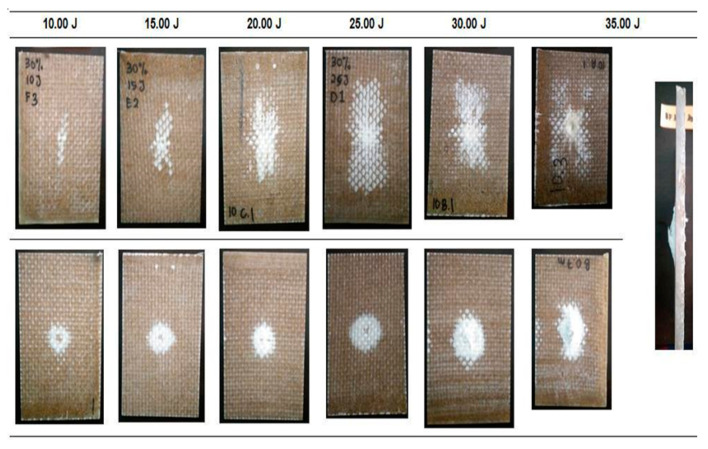
Visible damage on the impacted samples of the EP/G-BF30.

Figure 11 and Figure 12 show the damage propagation on the hybrid EP/G-BF10 and on the EP/G-BF30, respectively. Dye penetrant was not used as the damage is clearly visible and the area can directly be calculated from the surface of the impacted samples.

Generally, the damaged areas on both surfaces, top and bottom, were seen to increase as the impact energy level increased. A significant difference in the area was clearly observed on the top surfaces, while on the bottom perforation was detected [24,26]. Compared to the non-hybrid EP-BFC, the EP/G-BFC samples did not break into pieces during the full penetration event. The inclusion of woven glass fibres improved the properties of the composites in terms of impact damage resistance. This improvement is very beneficial for real life applications as the severity of failure is lower when compared to the total failure in the non-hybrid composites. The woven glass fibres can hold the structure in one piece during the highest impact incident. The damage area of the hybrid EP/G-BFC is presented in Table 7.

For both types of samples, EP/G-BF10 and EP/G-BF30, the damage area increased significantly from 10 J to 20 J, and a smaller difference was calculated as the energy level increased further. From 20 J to 35 J, the damage was seen to propagate more towards the bottom surface compared to the propagation from the centre to the sides of the samples.

## 4. Conclusions

The greater the peak force, the stiffer the projectile-to-target interaction, thus shortening the contact period of the impactor on the surface of the composites. The non-hybrid EP-BF10 composites have a stiffer projectile-to-target interaction compared to the EP-BF30 composites. However, neat epoxy samples showed the stiffest projectile-to-target interaction among the three samples, EP, EP-BF10, EP-BF30, at all impact energy levels. The non-hybrid EP-BF10 composites exhibited good stiffness but lower strength, while the EP-BF30 composites showed the opposite relation. Damage initiation and propagation in the EP-BF30 composites was slower and less severe when compared to the EP-BF10 composites. The non-visible damage in the bamboo composites, which occurred after the low velocity impact, can be analysed through the force undulations in the force-time graphs and can be observed using the dye penetrant method.

A significant improvement was observed with the inclusion of woven glass fibres in the composites. The non-hybrid composites broke into pieces during the highest impact energy that is applied, while the hybrid composites experienced only perforation and the structure did not totally break. The distance of the matrix cracking was shorter for the EP-BF30 when compared to the EP-BF10 at the same impact energy level, suggesting that increasing the bamboo fibre loading can improve the impact resistance, although in short fibre-reinforced composites.

## Figures and Tables

**Table 1:** Different impact energies applied to the EP, EP-BF composites and EP/G-BF composites.

Materials	Epoxy Matrix	E-Glass Fibre
**Density (g/cm^3^)**	1.10	2.58
**Tensile Strength (MPa)**	54	3445
**Tensile Modulus (GPa)**	3.2	72.3

**Table 2:** Different impact energies applied on the EP, EP-BF composites and EP/G-BF composites.

Composites	EP and EP-BF Composites					EP/G-BF Composites					
**Impact Energy (J)**	2.50	3.75	4.40	5.0	10.0	10.0	15.0	20.0	25.0	30.0	35.0

Different ranges of impact energy were applied to the non-hybrid and hybrid composites so as to achieve the maximum impact energy withstood by each type of composite.

**Table 3:** Data analysed from the force displacement graphs of EP, EP-BF10 and EP-BF30 at the first range of the impact energy level.

Sample	Impact Energy (J)	Peak Force (kN)	Energy Absorbed (J)	Peak Deformation (mm)
**EP**	2.50	1.31 (0.03)	0.85 (0.01)	4.16 (0.05)
	3.75	1.46 (0.02)	1.35 (0.02)	5.19 (0.05)
	4.40	1.57 (0.02)	1.61 (0.02)	5.51 (0.05)
	5.00	1.78 (0.03)	1.78 (0.03)	5.45 (0.03)
	10.00	2.57 (0.02)	4.72 (0.04)	7.23 (0.05)
**EP-BF10**	2.50	1.14 (0.01)	2.12 (0.04)	4.88 (0.03)
	3.75	0.65 (0.02)	3.32 (0.03)	9.22 (0.04)
	4.40	1.02 (0.04)	2.70 (0.03)	6.94 (0.04)
	5.00	-	-	-
	10.00	-	-	-
**EP-BF30**	2.50	0.91 (0.02)	1.76 (0.03)	3.99 (0.02)
	3.75	1.42 (0.03)	2.39 (0.05)	3.99 (0.03)
	4.40	0.98 (0.02)	2.95 (0.02)	6.43 (0.02)
	5.00	1.11 (0.02)	3.21 (0.02)	5.93 (0.05)
	10.00	-	-	-

**Table 4:** Data analysed from the force displacement graphs of EP/G-BF10 and EP/G-BF30 at the second range of the impact energy level.

Sample	Impact Energy (J)	Peak Force (kN)	Energy Absorbed (J)	Peak Deformation (mm)
**EP/G-BF10**	10	3.54 (0.04)	5.01 (0.03)	4.91 (0.04)
	15	3.78 (0.05)	9.29 (0.04)	6.41 (0.5)
	20	4.28 (0.05)	12.80 (0.07)	7.63 (0.05)
	25	4.87 (0.06)	17.05 (0.07)	8.53 (0.04)
	30	4.97 (0.05)	21.42 (0.07)	9.87 (0.05)
	35	5.24 (0.04)	27.92 (0.05)	11.32 (0.07)
**EP/G-BF30**	10	3.99 (0.03)	5.02 (0.05)	4.01 (0.05)
	15	4.55 (0.06)	8.10 (0.05)	4.89 (0.05)
	20	5.04 (0.05)	11.03 (0.04)	5.97 (0.04)
	25	5.55 (0.04)	14.85 (0.07)	6.63 (0.04)
	30	5.73 (0.05)	20.26 (0.05)	7.83 (0.06)
	35	6.10 (0.05)	25.87 (0.08)	8.96 (0.08)

**Table 5:** Distance travelled by the matrix cracking on the bottom surface of the EP-BF10 sample at different impact energy levels.

**Energy Levels (J)**	2.50	3.75	4.40	5.00
**Distance (mm)**	53	61	70	break

**Table 6:** Distance travelled by the matrix cracking on the bottom surface of EP-BF30 at different impact energy levels.

**Energy Levels (J)**	2.50	3.75	4.40	5.00	10.00
**Distance (mm)**	27	40	59	63	*break*

**Table 7:** Damage area for EP/G-BF10 and EP/G-BF30 at different energy levels.

**Energy Levels (J)**		10	15	20	25	30	35
**Area** **(mm^2^)**	**EP/G-BF10**	730	1700	3800	5500	5500	5600
	**EP/G-BF30**	300	700	1760	4000	3300	1000
